# Can trials of spatial repellents be used to estimate mosquito movement?

**DOI:** 10.1186/s13071-019-3662-x

**Published:** 2019-09-03

**Authors:** Josephine Malinga, Marta Maia, Sarah Moore, Amanda Ross

**Affiliations:** 1 Swiss Tropical and Public Health Institute, Basel, Switzerland; 2 University of Basel, Basel, Switzerland; 3 KEMRI Wellcome Trust Research Programme, Kilifi, Kenya; 4 Centre for Tropical Medicine and Global Health, University of Oxford, Oxford, UK; 5 Ifakara Health Institute, Ifakara, Tanzania

**Keywords:** Malaria, Mosquito movement, Spatial Repellent, *Plasmodium falciparum*

## Abstract

**Background:**

Knowledge of mosquito movement would aid the design of effective intervention strategies against malaria. However, data on mosquito movement through mark-recapture or genetics studies are challenging to collect, and so are not available for many sites. An additional source of information may come from secondary analyses of data from trials of repellents where household mosquito densities are collected. Using the study design of published trials, we developed a statistical model which can be used to estimate the movement between houses for mosquitoes displaced by a spatial repellent. The method uses information on the different distributions of mosquitoes between houses when no households are using spatial repellents compared to when there is incomplete coverage. The parameters to be estimated are the proportion of mosquitoes repelled, the proportion of those repelled that go to another house and the mean distance of movement between houses. Estimation is by maximum likelihood.

**Results:**

We evaluated the method using simulation and found that data on the seasonal pattern of mosquito densities were required, which could be additionally collected during a trial. The method was able to provide accurate estimates from simulated data, except when the setting has few mosquitoes overall, few repelled, or the coverage with spatial repellent is low. The trial that motivated our analysis was found to have too few mosquitoes caught and repelled for our method to provide accurate results.

**Conclusions:**

We propose that the method could be used as a secondary analysis of trial data to gain estimates of mosquito movement in the presence of repellents for trials with sufficient numbers of mosquitoes caught and repelled and with coverage levels which allow sufficient numbers of houses with and without repellent. Estimates from this method may supplement those from mark-release-recapture studies, and be used in designing effective malaria intervention strategies, parameterizing mathematical models and in designing trials of vector control interventions.

## Background

There has been an increase in interest in the movement of vectors and people, and how each contributes to the spread of malaria infections [1–4]. The flow of infections within and between households in an area has implications for interventions such as targeting areas or transmission foci and reactive case detection [5, 6]. Mosquito movement is the main mode of spread of malaria parasites within a community. Hence, information on how mosquitoes move can help inform the design of intervention strategies and aid in the parameterization of mathematical models to predict their likely impact [7]. It can also inform the design of cluster-randomised controlled trials (cRCTs) to estimate the effect of new tools [8].

There is limited information on the movement of mosquitoes between households. Vector dispersal has been estimated at different spatial and temporal scales using mosquito mark-release-recapture (MMRR) and genetic markers [9–14]. Previous MMRR studies have shown that approximately 80% or more of mosquitoes recaptured are within three kilometres of release points up to two weeks after release [7, 15–17], including those emerging from breeding sites [18–20]. Long-range movement between villages, or farther, is only occasionally observed [17, 21, 22]. Distances moved by the mosquito vary by vector species, distribution of host habitats, wind direction and the use of vector control interventions [15, 17, 18] among other factors.

Both MMRR and genetic methods have limitations. MMRR is dependent on the recapture success, which is affected by factors ranging from geographical landscapes and climate, vector population structure and behaviour, collection effort as a function of distance from release points [10] and how systematic the sampling is, in addition to ethical implications regarding the release of potential disease vectors back into the environment. Population genetic studies using microsatellites and other molecular variants to define fine-scale genetic patterns of vectors might be subject to resolution effects, masking patterns [12], and are very costly, limiting the number of mosquitoes that can be analysed. These studies are challenging to carry out and further sources of data would be valuable.

A potential source of data on mosquito movement which has not been fully harnessed is trials of repellents. To estimate the effect of topical and spatial repellents, mosquito densities in households with and without repellents [23–32] have been compared. Some studies have estimated the extent to which mosquitoes are diverted to houses without repellents when there is less than full coverage in a study area. They reported the possibility of diversion to non-users [31, 33], no change in mosquito densities collected [33, 34], while some experimental trials outlined the impact of the repellents on the mosquito olfactory cues and delayed feeding [35, 36]. These studies have not estimated the distance between households that the mosquitoes were diverted. We sought to determine if data from the trials with diversion could be used to estimate fine-scale movement of mosquitoes in the presence of spatial repellents as a secondary analysis and whether modifications to the trial design would be necessary to achieve this.

Mosquito movement is likely to be altered by the presence of repellents. Spatial repellents such as transfluthrin induce orthokinesis, where the mosquito moves in a random fashion until it moves into a lower concentration when it resumes natural flight [36, 37]. Therefore, estimates of mosquito movement in the presence of spatial repellents complement those from other data sources.

We developed a statistical model for estimating the movement of mosquitoes that are repelled. We validated the model using simulation to determine the characteristics of a study under which the model could provide accurate estimates of the parameter values. We applied the model to observed data from a trial in Tanzania where the main objective was to investigate whether mosquitoes are diverted from users to non-users of spatial repellents in an area of residual transmission and incomplete spatial repellent coverage [33].

## Methods

### Trial design

We use a trial of spatial repellents from Tanzania described previously in Maia et al. [33]. Briefly, the study was conducted in three villages, each with 30 households. The distance between any two villages was greater than two kilometres while households within villages were on average within 0.1 km to 0.3 km of each other. The study took place over 24 weeks between December 2012 and June 2013. Three coverage scenarios with mosquito coils containing 0.03% transfluthrin were rotated every two weeks among the villages: (i) 100% coverage; (ii) no coverage; (iii) incomplete coverage with 80% of the households using coils. Coils were distributed and used on each day of the week. Blank coils were used as a placebo. Mosquitoes were collected outdoors under the kitchen thatch roof as well as indoors for three consecutive days each week using Prokopok aspirating devices [38]. There were a total of 72 collection days from each household. The presence of a spatial repellent in a household was defined as a combination of two features, availability of a coil with transfluthrin, and observed compliance to coil use. Compliance was assessed by inspecting the ashes produced the previous night.

The original study compared the numbers of mosquitoes collected in households in four groups: households using repellent in weeks with complete coverage in a village; households using repellent in a village when there was incomplete coverage; households not using repellent in a village when there was incomplete coverage; and households in a village when there was zero coverage. For our analysis, we use *Anopheles arabiensis* mosquitoes since they were the most repelled by the active coils.

### Statistical model

#### Model strategy

We developed a statistical model with the aim of estimating the geographical distances between households that the mosquitoes diverted by the repellent move from and to. Movement of individual mosquitoes cannot be determined, but we can estimate the population parameters such as the mean distance moved between houses.

**Fig. 1:**
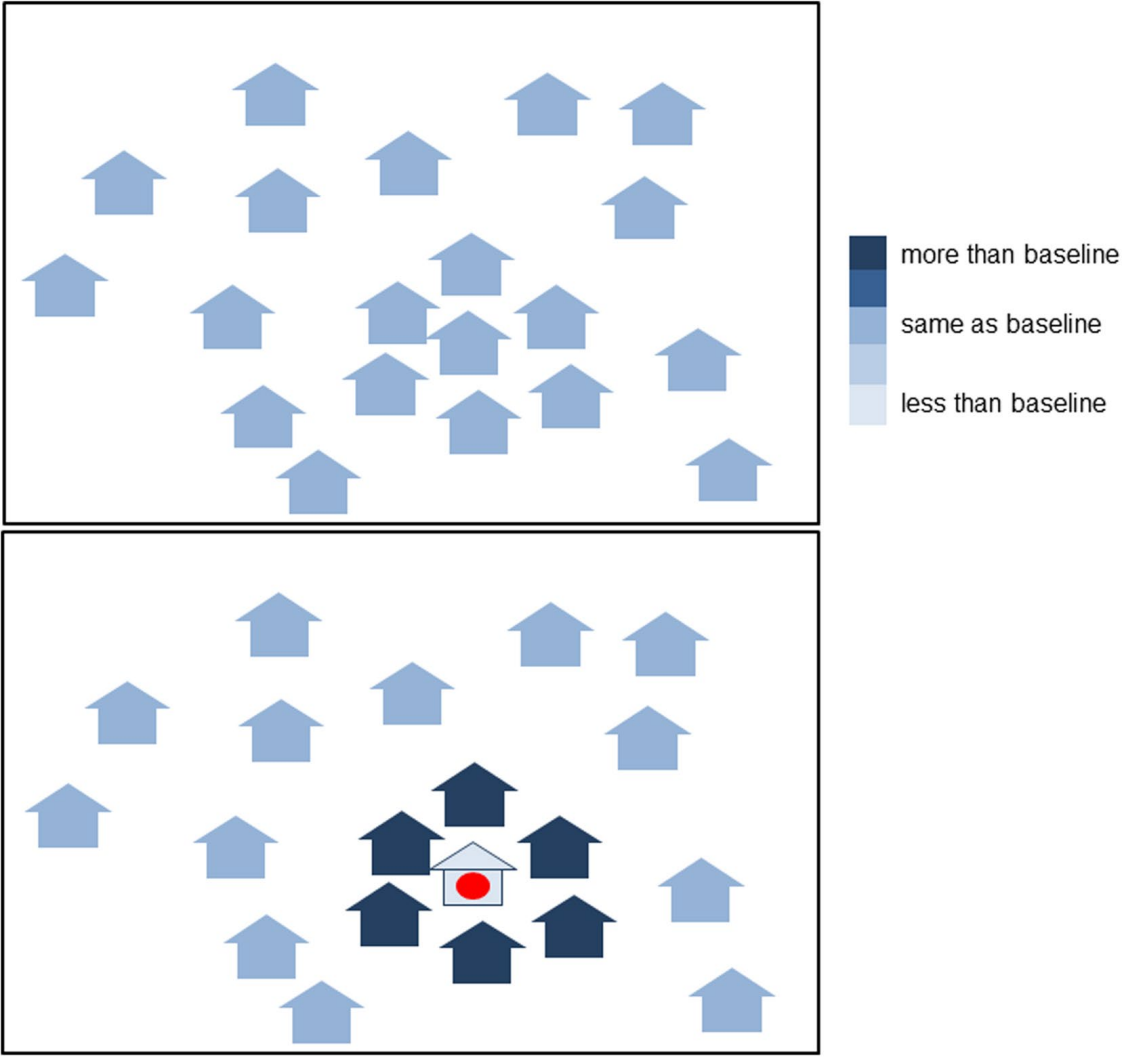
Schematic diagram of how the method works. Houses: proportion of the total number of mosquitoes in each house relative to the proportion when there is zero coverage. Upper panel: with zero coverage. The distribution of mosquitoes between houses in the village when there is zero coverage is the baseline distribution. The houses may differ in mosquito density. The total number of mosquitoes in the village may vary over time, but it is assumed that the relative proportions in each house stay constant. Lower panel: A coil with spatial repellent is used in the house with the red circle. If the repellent diverts mosquitoes, then the number of mosquitoes in this house will decrease and the number in the surrounding houses may increase. The share of the total number of mosquitoes in each house will differ from the baseline distribution. The model uses the differences in the distribution of mosquitoes between houses for varying coverage levels in the trial to estimate parameters of mosquito movement

We defined the baseline distribution of the proportion of mosquitoes in each house as the distribution of mosquitoes when there is 0% coverage. The proportions may vary between houses and must sum to one. The total number of mosquitoes per day can vary throughout the study period but we assumed that the proportions in each house remain the same in the absence of repellent use. In the case of unfed mosquitoes emerging from breeding sites, this assumption is unlikely to be true. Seasonal patterns in rainfall may vary emergence rates from breeding sites, and newly emerged mosquitoes may cluster in houses closest to a breeding site. Therefore, we restricted the analysis to blood-fed mosquitoes only. Mosquitoes in general take a few days for their first blood meal [39, 40] allowing time for dispersal away from the breeding site. We assumed that the distributions of mosquito densities which differ from the baseline distribution when a proportion of households use repellent reflect the movement of the diverted mosquitoes (Fig. 1).

The model derives the expected proportion of mosquitoes in each house based on the baseline distribution of mosquitoes between houses when there is zero coverage and the excess outgoing and incoming mosquitoes for each house when some households use spatial repellents. The parameters that govern the outgoing and incoming mosquitoes to be estimated are, the proportion of mosquitoes diverted when repellent is used ($\beta$), the mean distance between households moved by the diverted mosquitoes (λ), and the proportion of those diverted that go to another house as opposed to elsewhere such as vegetation ($\varphi$).

Model A is the base model. Model B is a small extension of model A in the case where data on the seasonal pattern of mosquito densities in the absence of spatial repellents are available.

#### Model A

Let $N_{t}$ be the total number of mosquitoes collected from all households on day $t$. We assume that in the 0% coverage scenario, the proportion of mosquitoes in each house $h$ in a village is given by $C_{1} , C_{2} \ldots  C_{h}$.

The proportion of mosquitoes diverted by the repellent is represented by $$ \beta $$. We use $$ a $$ and $$ b $$ to denote the house that a mosquito is potentially diverted from and to. Of the total number of mosquitoes in houses on day $$ t $$, $$ N_{t} $$, the proportion diverted from house $$ a $$, $$ O_{at} , $$is given by the proportion in the house in the absence of intervention$$ ,  C_{a} , $$ multiplied by the proportion diverted, $$ \beta , $$ so that

1 $$ O_{at} = C_{a}  \beta  s_{at} $$

where $s_{at}$ is equal to 1 in a house with repellent use on that day and 0 if the repellent was not used. $O_{at} = zero$ if no spatial repellent was used.

Diverted mosquitoes may move to another house with probability or to somewhere outside the houses with probability $$ \left( {1 - \varphi } \right) $$. Conditional on moving from house $$ a $$ to another house, the probability that a mosquito moves to house $$ b, $$ $$ \Pr \left( {M_{abt} |M_{a.t} } \right), $$ depends on a function $$ f $$ of the distance in kilometres, $$ d_{ab} , $$ and the repellent status in house $$ b $$ on day $$ t $$. The probability is scaled so that the probabilities of moving to each house in the village, conditional on moving to a house, sum to one.

2 $$ Pr\left( {M_{abt}  | M_{a.t} } \right) = \frac{{f\left( {d_{ab} } \right) s_{bt} }}{{\mathop \sum \nolimits_{b} f\left( {d_{ab} } \right) s_{bt} }} $$

**Fig. 2:**
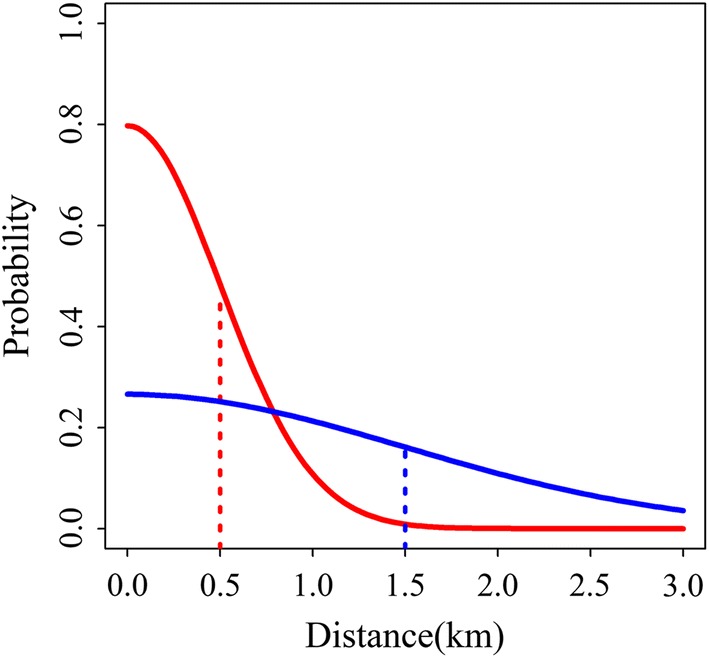
Distributions of geographical distances using the normal kernel corresponding to mean distance values. Red line: the mean, *λ*, is 0.5 km; blue line: *λ* is 1.5 km

We set the function $f$, which describes the chance of the mosquito moving to a house depending on distance, to a normal kernel (Fig. 2), which is similar to diffusion and represents the distance travelled by a random walk in a fixed time period, so that,

3 $$ f\left( {d_{ab} } \right) = \exp \left( { - \frac{1}{2} \lambda^{ - 2} d_{ab}^{2} } \right) $$

where *λ*, the mean distance, is to be estimated. Other distributions may also be used.

The proportion of all mosquitoes on day $$ t $$ who are diverted to house $$ b $$ from house $$ a $$, $$ I_{abt} , $$ is given by multiplying the probability for being diverted from house $$ a $$ given repellent use, $$ O_{at} $$, the probability of being diverted to a house rather than elsewhere, $$ \varphi $$, and the conditional probability of moving to house $$ b $$ given that the mosquito has moved to another house from house $$ a $$ so that,

4 $$ I_{abt} = Pr\left( {M_{abt}  | M_{a.t} } \right) \varphi  O_{at} $$

The proportion moving to house $$ b $$ from all other houses, $$ I_{bt} , $$ is then summed over all houses,

5 $$ I_{bt} = \mathop \sum \limits_{a } I_{abt} $$

The proportion of mosquitoes in house $$ h $$ of all mosquitoes on day$$ t $$, $$ P_{ht} , $$ is then given by the baseline proportion (that would occur if there is zero coverage), $$ C_{h} , $$ minus the proportion of diverted mosquitoes, $$ O_{ht} , $$ and adding the proportion of incoming mosquitoes, $$ I_{ht} , $$

6 $$ P_{ht} =  C_{h} - O_{ht} + I_{ht} $$

The observed densities, $$ Y_{ht} , $$ are based on mosquitoes in houses only (as opposed to those diverted elsewhere) and include houses with missing data. To correspond to the observed densities, we set the predicted proportions to zero in houses with missing mosquito densities. This makes no assumption about their actual values. We then re-scale the proportions to sum to one.

7 $$ Q_{ht } = \frac{{ P_{ht}  w_{ht} }}{{\mathop \sum \nolimits_{h}  P_{ht}  w_{ht} }} $$

where $w_{ht}$ is an indicator set to 0 if the house has a missing mosquito density on that day and 1 if the data are present.

The observed densities follow a multinomial distribution around the predicted probabilities.

8 $$ Y_{ht} \sim Mn\left( {Q_{1t} , \ldots ,Q_{Ht} , N_{t} } \right) $$

#### Model B

If data on the total number of mosquitoes including those diverted elsewhere are available, then there is potentially more information with which to disentangle the effects of repellency, movement and diversion elsewhere. Mosquitoes diverted elsewhere are not sampled in the houses but this information can be gained by having data on the seasonal pattern of mosquito densities in the absence of spatial repellents, either from another control village or from a rotation of coverage levels which allows the seasonal pattern to be estimated.

The model can be modified to incorporate information on the proportion of mosquitoes that are diverted elsewhere. In this case, the observed data are fitted to a multinomial distribution with the expected proportion in each house, $Q_{ht}^{\prime}$ and an additional category for the expected proportion of mosquitoes that were diverted elsewhere, $Q_{et}^{\prime} .$ These probabilities are scaled to sum to one. The total number of mosquitoes is $N_{t}^{\prime}$.

9 $$ Y_{ht} \sim Mn\left( {Q_{1t}^{\prime} , \ldots , Q_{Ht}^{\prime} , Q_{et}^{\prime} , N_{t}^{\prime} } \right) $$

where $Q_{et}^{\prime}$ is calculated by subtracting $N_{t}$ (the total number of mosquitoes sampled from households) from $N_{t}^{\prime}$ (total number of mosquitoes including those which are diverted elsewhere).

#### Quantities in the models

The quantities in the models are shown in Table 1.

**Table 1:** Quantities in the models

Quantity	Description
Included in model A and model B	
$N_{t}$	Number of mosquitoes caught in all houses on day $t$
$C_{h}$	Baseline proportion of mosquitoes in house $h$ of mosquitoes in all houses, when there is zero coverage
$P_{ht}$	Proportion of mosquitoes in house $h$ on day $t$ of those in all houses
$O_{at}$	Proportion of mosquitoes diverted from house $a$ on day $t$ of those in all houses
$I_{bt}$	Proportion of mosquitoes diverted to house $b$ on day $t$ of those in all houses
$Pr(M_{a.t} )$	Probability of mosquito being diverted from house $a$ on day $t$
$Pr(M_{abt} )$	Probability that diverted mosquitoes move from house $a$ to house $b$
$Q_{ht }$	Predicted proportion of mosquitoes in house $h$ on day $t$ of those in all houses
$\beta$	Proportion of mosquitoes diverted of those in houses using repellent
$\varphi$	Proportion of mosquitoes moving to another house of those diverted
${{\uplambda }}$	Mean distance between households for diverted mosquitoes
$s_{at}$	Presence of spatial repellent in house $a$ on day $t$
$d_{ab}$	Distance between house $a$ and house $b$
Additionally included in model B	
$N_{t}^{'}$	Number of mosquitoes caught in houses and those diverted elsewhere
$Q_{et}^{'}$	Predicted proportion of mosquitoes diverted elsewhere on day $t$ of mosquitoes in houses or diverted elsewhere
$Q_{ht}^{'}$	Predicted proportion of mosquitoes in house $h$ on day $t$ of those in all houses or diverted elsewhere

### Implementation

The statistical model was written in C++. The simulations were run on sciCORE (http://scicore.unibas.ch/) scientific computing core facility at the University of Basel. We used Nelder–Mead optimization [41, 42] to maximize the multinomial log-likelihood in order to estimate the parameters of interest; the proportion of mosquitoes repelled from houses with spatial repellents ($\beta$), the mean distance between households moved by the diverted mosquitoes (*λ*), and the proportion of mosquitoes repelled that go to a household as opposed to elsewhere ($\varphi$). The code is available at https://github.com/SwissTPH/mosquito-movement-spatial-repellent-method.

### Model validation

We evaluated the ability of the models to recover known values using simulated data. We assessed the method under different conditions to establish at what level of coverage, the proportion of mosquitoes repelled from households using repellents, the proportion of mosquitoes repelled going to households as opposed to elsewhere, and mean total number of mosquitoes collected per day, the model is able to reproduce accurate parameter values.

We base the scenarios of trial characteristics on the design of the trial of spatial repellents from Tanzania. We specified a reference scenario in which the model could work well and varied each of the input parameters in turn to determine the values at which the model no longer works well (Table 2). We simulated trial datasets of observed numbers of fed mosquitoes for each household per day using our underlying model assumptions and random variation. Since there is stochasticity in the simulations, we simulated 100 datasets for each scenario, and estimated the parameter values for each dataset.

**Table 2:** Simulated scenarios of trial characteristics to evaluate the method

Quantity	Value	Source
$\beta ,$ proportion diverted from houses using repellent	0.10, 0.30, **0.50**, 0.80	To be estimated
$\varphi ,$ proportion of those diverted that go to another house	0.20, **0.50**, 0.80	To be estimated
$\lambda$, mean distance of movement for diverted mosquitoes (km)	0.05,**0.20**,0.30,0.50,0.80	To be estimated
$$ N_{t} , $$ number of mosquitoes on day $$ t $$ in the houses (Model A)	10,100, 1000 (mean of 0.3, 3, 30 mosquitoes per house)	Given by dataset
$$ N_{t}^{'} , $$ number of mosquitoes on day $$ t $$ including those diverted elsewhere (Model B)	10,100, **1000**	Estimated from additional data on seasonal pattern of mosquito densities
Number of experimental days	72	Trial characteristic input
Number of days with zero coverage	18	Trial characteristic input
Number of households with spatial repellent out of 30 per day	6, **15**, 24, 28	Trial characteristic input

*Note*: The reference scenario is indicated by bold font

For simplicity, the total number of mosquitoes collected per day remains constant and there is no seasonality.

## Results

### Trial data

The trial characteristics for the three villages are summarized in Table 3.

**Table 3:** Trial characteristics for the three villages

	Uwata	Matete	Igima
Number of mosquitoes collected per day			
Median (90% central range)	6 (2–20)	2 (0–9)	0 (0–3)
Distance between all pairs of households (km)			
Median (90% central range)	0.31 (0.14–0.50)	0.21 (0.07–0.30)	0.14 (0.09–0.21)
Compliance to repellent use in each treatment arm			
Complete coverage	90%	89%	93%
Incomplete coverage^a^	90%	90%	93%

^a^The denominator is the total number of households allocated the treatment. There were 30 households in each study village

### Model validation

We used simulation to assess how well the method was able to recover known parameter values. The simulations are based on the design of the trial and using the house coordinates for one village, Uwata.

**Fig. 3:**
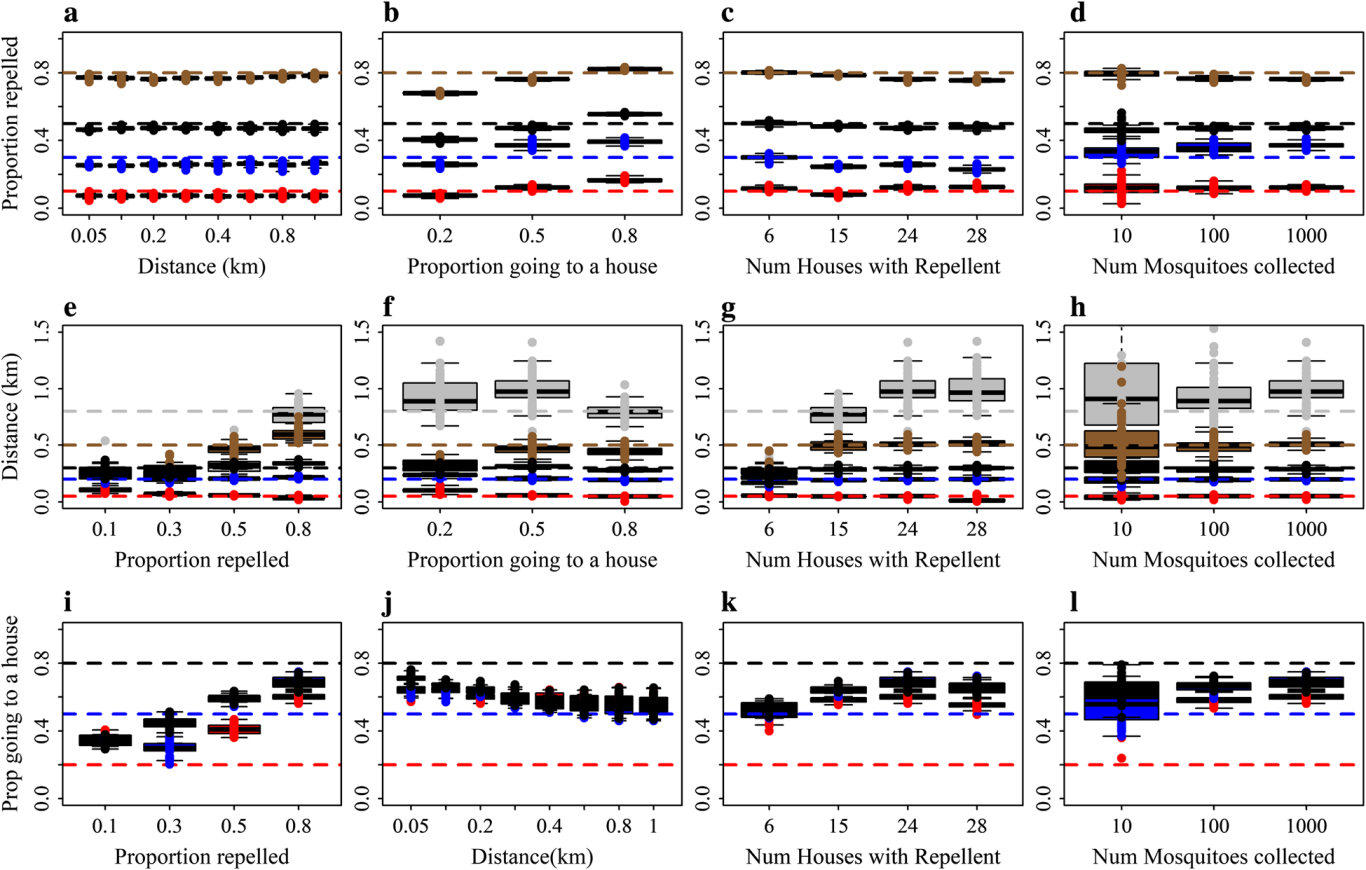
The ability of Model A to return known parameter values. Estimated values for $\beta$, the proportion of mosquitoes repelled from houses using spatial repellents (top row). **a**-**d** Estimates by mean distance between households moved by diverted mosquitoes (**a**), by proportion of mosquitoes repelled that go to households as opposed to elsewhere (**b**), by the number of households using spatial repellents (out of the total of 30) (**c**), and by the total number of mosquitoes collected from all households per day (**d**). The horizontal lines represent the different known values to be returned coded by colour: red (0.1); blue (0.3); black (0.5); and brown (0.8). **e**-**h** Estimated values for *λ*, the mean distance between households moved by diverted mosquitoes (middle row) by the proportion of mosquitoes repelled from houses using a spatial repellent (**e**), by the proportion of mosquitoes repelled that go to households as opposed to elsewhere (**f**), by the number of households using spatial repellents out of a total of 30 (**g**); and by the total number of mosquitoes collected per day from all households (**h**). The horizontal lines represent the different known values to be returned coded by colour: red (0.05 km); blue (0.2 km); black (0.3 km); brown (0.5 km); and grey (0.8 km). **i**–**l** Estimated values for $\varphi$, the proportion of mosquitoes repelled that go to households as opposed to elsewhere (bottom row) by the proportion of mosquitoes repelled (i), by the mean distance between households moved by diverted mosquitoes (**j**); by the number of households using spatial repellents on any given day (**k**); and by the total number of mosquitoes collected from all households per day (l). The horizontal lines represent the different known values to be returned coded by colour: red (0.2); blue (0.5); and black (0.8). The boxplots represent the estimated values from 100 simulated datasets for each scenario. The reference scenario is based on the trial design and trial house coordinates and is given in Table 2. We alter one characteristic at a time

Model A worked well for some parameters, but not others. The estimates are reasonable for $\beta$, the proportion of mosquitoes repelled from households with repellents, across the range of values for distance (Fig. 3a), the proportion of diverted mosquitoes that go to another house (Fig. 3b), for different coverage levels (Fig. 3c), and numbers of mosquitoes collected per day (Fig. 3d). However, for $\lambda$, the mean distance of diversion between houses, model A estimates were accurate only for scenarios where 80% of mosquitoes were repelled (Fig. 3e), 80% of mosquitoes repelled went to a house (Fig. 3f) and with a coverage of around 50% (Fig. 3g). The estimates for $\varphi$, the proportion of mosquitoes repelled that go to households as opposed to elsewhere were poor for all scenarios (Fig. 3, bottom row).

For Model A, there was little information in the simulated datasets to disentangle the effects of the mean distance moved by mosquitoes that were repelled, the proportion repelled and the proportion of mosquitoes repelled that go to households as opposed to elsewhere.

For Model B, we extended the model to include data on mosquitoes that were diverted elsewhere. For the method evaluation, this can be simulated easily but for a trial, data on the seasonal pattern in the absence of repellents would be required.

**Fig. 4:**
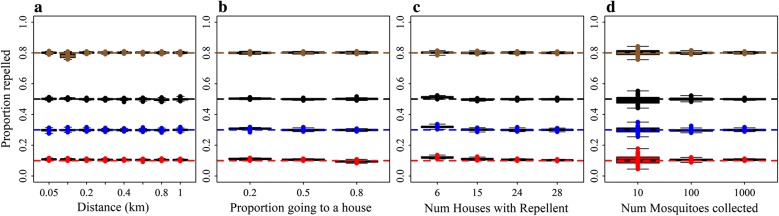
The ability of Model B to return the known values for $\beta ,$ the proportion of mosquitoes repelled from houses using spatial repellents. **a** By mean distance between households moved by diverted mosquitoes. **b** By proportion of mosquitoes repelled that go to households as opposed to elsewhere. **c** By the number of households using spatial repellents (out of the total of 30). **d** By the total number of mosquitoes collected from all households per day. The horizontal lines represent the different known values to be returned coded by colour: red (0.1); blue (0.3); black (0.5); and grey (0.8). The boxplots represent the estimated values from 100 simulated datasets for each scenario. The reference scenario is based on the trial design and trial house coordinates and is given in Table 2. We alter one characteristic at a time

The model returned the correct values for $\beta$, the proportion of mosquitoes repelled from households with spatial repellents, for all levels assessed for the mean distance between households (Fig. 4a), the proportion of mosquitoes repelled that go to households as opposed to elsewhere (Fig. 4b), the coverage of households using spatial repellents (Fig. 4c) and the total number of mosquitoes collected per day (Fig. 4d).

**Fig. 5:**
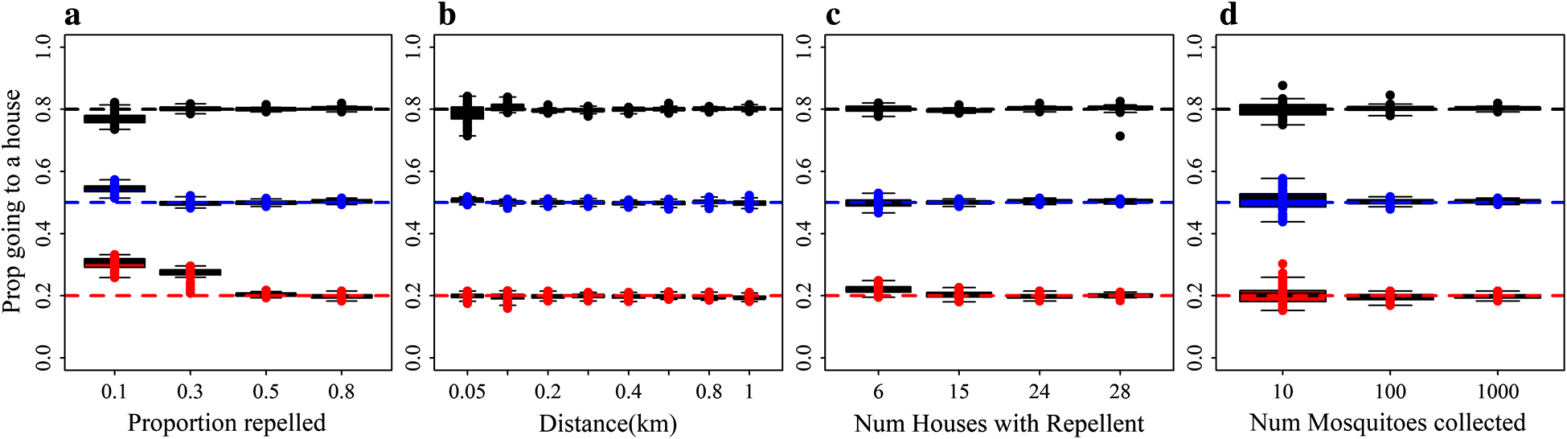
The ability of Model B to return known values for $\varphi$, the proportion of mosquitoes repelled that go to households as opposed to elsewhere. **a** By the proportion of mosquitoes repelled. **b** By the mean distance between households moved by diverted mosquitoes. **c** By the number of households using spatial repellents on any given day. **d** By the total number of mosquitoes collected from all households per day. The horizontal lines represent the different known values to be returned coded by colour: red (0.2); blue (0.5); and black (0.8). The boxplots represent the estimated values from 100 simulated datasets for each scenario. The reference scenario is based on the trial design and trial house coordinates and is given in Table 2. We alter one characteristic at a time

Estimates for $\varphi$, the proportion of mosquitoes repelled that go to households as opposed to elsewhere, were also reproduced precisely over the range of mean distance (Fig. 5b), coverage (Fig. 5c) and total number of mosquitoes collected per day (Fig. 5d). But, estimates were less precise if the proportion of mosquitoes repelled was low (Fig. 5a). There was too little information provided by the relatively small number of mosquitoes repelled to produce accurate estimates.

#### Mean distance between households moved by the mosquito

**Fig. 6:**
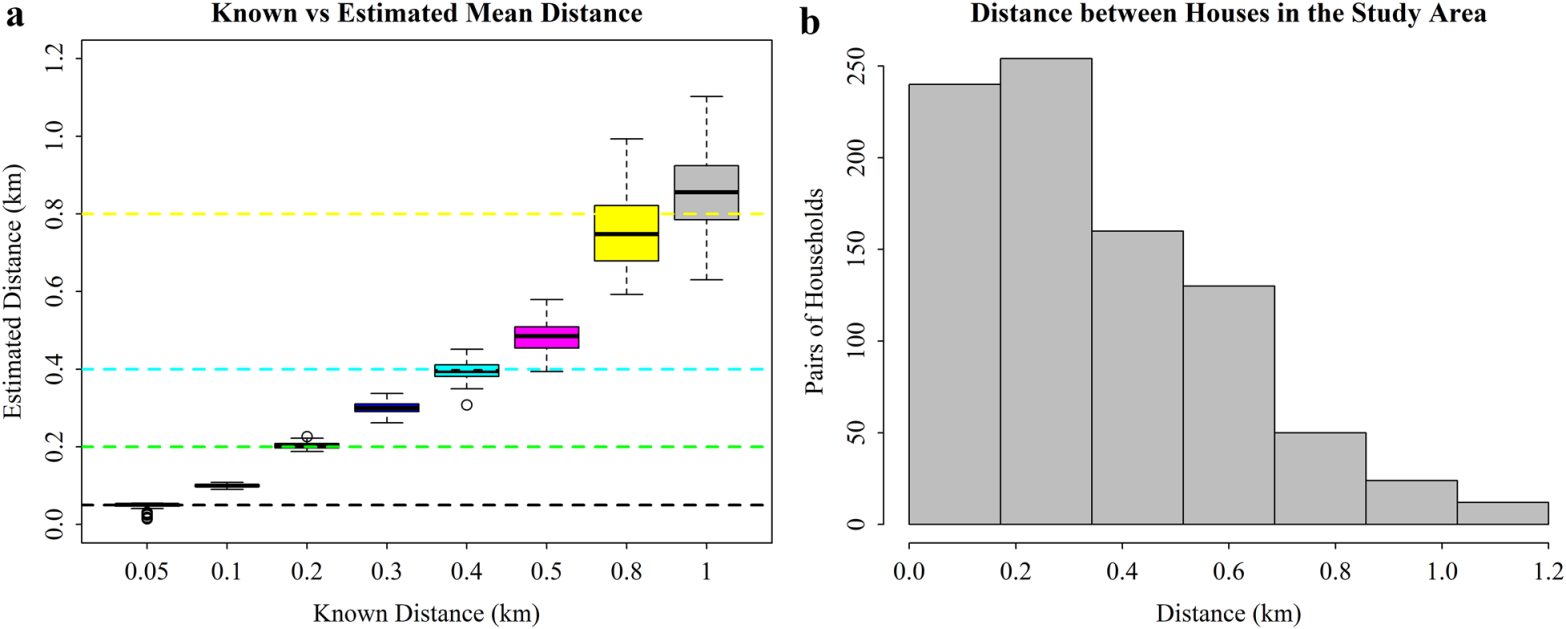
**a** Estimated (box plots) and actual (dotted lines) simulated mean distances between households moved by mosquitoes diverted by the spatial repellent. **b** Distribution of the distances between all pairs of households in the study area. The boxplots in **a** represent the estimated values from 100 simulated datasets for each scenario, and the colours represent the different mean distances. The reference scenario is based on the trial design and trial house coordinates and is given in Table 2

Estimates for *λ*, the mean distance between households that the mosquitoes were diverted, were accurate when the known values were shorter, but not when the mean was greater than 0.8 km (Fig. 6a). This is likely to be due to the configuration of the trial village where more than 90% of distances between pairs of houses were less than 800 m apart (Fig. 6b).

**Fig. 7:**
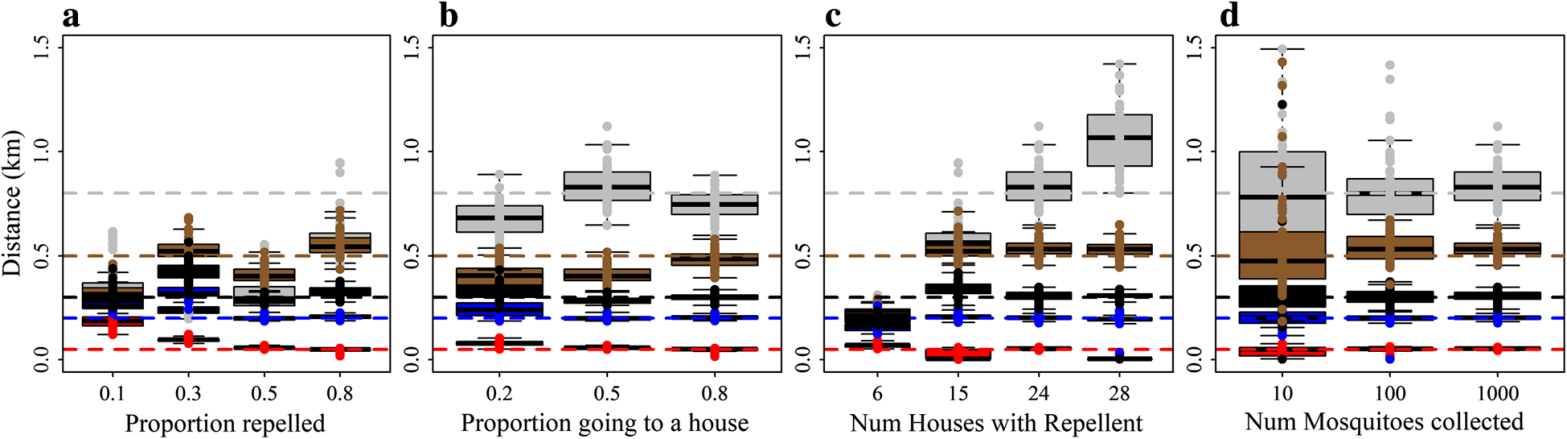
The ability of Model B to return known values for *λ*, the mean distance between households moved by mosquitoes. **a** By the proportion of mosquitoes repelled from houses using a spatial repellent. **b** By the proportion of mosquitoes repelled that go to households as opposed to elsewhere. **c** By the number of households using spatial repellents out of a total of 30. **d** By the total number of mosquitoes collected per day from all households. The horizontal lines represent the different known values to be returned coded by colour: red (0.05 km); blue (0.2 km); black (0.3 km); brown (0.5 km); and grey (0.8 km). The boxplots represent the estimated values from 100 simulated datasets for each scenario. The reference scenario is based on the trial design and trial house coordinates and is given in Table 2. We alter one characteristic at a time

For mean distances of 800 m or less, the model estimates were accurate for scenarios where more than 30% of mosquitoes were repelled (Fig. 7a), 50% or more mosquitoes repelled went to a house (Fig. 7b), and with sufficient coverage (Fig. 7c) and a higher number of mosquitoes collected per day (Fig. 7d). One hundred mosquitoes per day (3 per house) provided precise estimates, but for 10 (0.3 per house) the precision was less. If there is low coverage, or few mosquitoes are repelled, then there is little information in the dataset to estimate the mean distance.

### Application to data from Kilombero Valley, Tanzania

We applied the method to the observed trial data on collected mosquito densities from Kilombero, Tanzania (Table 3). The trial data have characteristics which, from the method evaluation, indicate that the model would not provide accurate estimates. The mosquito densities were low and there was a very low proportion of mosquitoes repelled. There was no evidence of an impact on mosquito abundance of the spatial repellent [33]. It has been suspected that the transfluthrin concentration in the coils might have been too low to repel mosquitoes in this particular study.

We applied the model to two of the villages, Uwata and Matete (Table 4). Due to the extremely low numbers of mosquitoes collected in Igima, it was excluded from further analysis. The estimates are consistent with the study findings that a very low proportion of mosquitoes were diverted by the spatial repellents (Table 4).

**Table 4:** Parameter estimates using the observed data

Parameter estimate^a^	Uwata estimate (95% CI)	Matete estimate (95% CI)
Proportion of mosquitoes repelled	0.04 (0.03–0.04)	0.04 (0.03–0.05)
Mean distance moved between households	0.12 (0.09–0.35)	0.04 (0.00–0.14)
Proportion of mosquitoes moving to households of those repelled	0.88 (0.54–1.00)	0.87 (0.04–1.00)
Log-likelihood	− 1716.51	− 686.02

^a^Blood-fed *Anopheles arabiensis* mosquitoes were used in this analysis

## Discussion

We developed a statistical model to estimate the proportion of mosquitoes repelled from households using spatial repellents, the proportion of those repelled that are diverted to another house and how far apart the houses are. The evaluation of the method suggests that although Model A, the model without information on seasonality, works well for some parameters, it does not provide accurate estimates for others. The method only works well for all parameters when there is information on the total number of mosquitoes in the study area, including those diverted elsewhere as opposed to only those diverted to households (Model B). Taken together, these results suggest that trials of repellents could potentially be used to estimate mosquito movement, as long as the trial design is modified so that information on the total number of mosquitoes or the seasonality pattern is available.

Findings from this study may help quantify the criteria for trial settings seeking to estimate mosquito movement by providing insights on what type of data needs to be collected. Our results show that trial data need to contain sufficient information for the different variables. We found that estimates were not precise if there was a low coverage with repellents (less than 50%) and a low proportion of mosquitoes were repelled from households using repellent (less or equal to 30%). Estimates for longer mean distances moved between households by the repelled mosquitoes (greater than 800 m) were also imprecise: this is expected since the houses in the village were closely arranged. Estimates were reasonably precise if 100 mosquitoes per day (3 per house) were caught, but less precise if this was reduced to 10 mosquitoes (0.3 per house). Simulation could be further exploited to refine the trial design, by investigating factors such as the number of mosquito collections and number of houses when specific trials are being planned.

Estimates of mosquito movement in the presence of interventions can inform the design of trials of interventions where effectiveness is affected by movement generally. Mosquito movement has been shown to affect the effectiveness of interventions: the effectiveness can be attenuated through contamination from different study arms, or community wide effects conferred to the surrounding areas [8]. In previous trials of bed nets in some African settings, the failure to observe any significant differences between the intervention and control study villages may be partially attributed to the movement of mosquitoes between villages which might have led to the underestimation of the intervention effect [8, 22]. In Tanzania, mosquitoes were diverted to non-users in trials of topical and spatial repellents in sites with incomplete coverage [31, 33], highlighting the need for feasible allotment strategies if complete coverage is hard to achieve. These estimates of mosquito movement can also be used to parameterize mathematical models for assessing the anticipated impacts of intervention strategies where data is not available. It is not clear how much mosquito movement varies depending on whether spatial repellents are present or absent, but as more studies are carried out, further estimates will become available and potentially allow comparison in similar settings.

The need to estimate mosquito movement from as many sources of data as possible stems from the low number of datasets designed specifically to measure mosquito movement. This is compounded by the need to have estimates from different settings and in the presence of different interventions due to the lack of generalizability. The distances travelled are highly dependent on the setting, due to factors like the vector species and environmental features such as vegetation, breeding sites, wind direction and the spatial distribution of households [13, 18]. Although the method developed does not work when there are low numbers of mosquitoes repelled as in the available dataset, it does work in other settings and can inform trial design for future studies.

There are some limitations with our modelling strategy. We did not take into account the number of consecutive evenings that the spatial repellent had been used within each two-week period of intervention or placebo but rather assumed that the effect was constant over time. This may not be correct, and could be validated by estimating any trend in mosquito densities among the houses over the fortnight in trials with sufficiently large numbers of mosquitoes. The model could be extended to take further time detail into account, for example by using the estimates of the previous day for the distribution of mosquitoes between houses as the baseline proportions of the current day. Validation could be carried out using further datasets and approaches such as individual-based simulation modelling of mosquito movement (Denz et al., unpublished data). Our model could also be extended to test hypotheses about mosquito movement, such as whether mosquitoes prefer to move to the first house they encounter without repellents or any other. Incorporating data on the number of hosts as a measure of attractiveness or on excess mosquito mortality could refine the estimates.

The low proportion of mosquitoes repelled from households using spatial repellent estimated by the method was consistent with results published previously [33], where there were no significant differences in the number of mosquitoes collected in households with and those without spatial repellents. The reason for the lack of repellency is likely to be the concentration of transfluthrin which would have been too low for substantial action in natural settings with free air movements [37]. The deterrence and repellency effects of transfluthrin are dose-dependent with substantial protective effects seen at higher concentrations than those used in the current study e.g. 0.03% transfluthrin coils used indoors [35]. Using blank coils as a placebo may also reduce differences between houses with spatial repellents and those without due to the effects of smoke.

## Conclusions

We developed a statistical model as a potential tool to gain information on mosquito movement from trials of repellents. If the design of trials of repellents is modified to provide information on the total number of mosquitoes using the seasonal pattern, then the method is able to reproduce known values from simulated datasets well. Further work to validate the method in field settings is needed. Estimates of mosquito movement can inform the design of both intervention strategies and trials of interventions where effectiveness is affected by movement generally, and in particular estimates of movement in the presence of spatial repellents may inform decisions on implementation and allocation.

## Data Availability

The data of the analysed trial are available from the authors upon request. The code is available at https://github.com/SwissTPH/mosquito-movement-spatial-repellent-method.
